# Ureterocele as differential diagnosis of hydrosalpinx—an interesting case from the clinical practice

**DOI:** 10.1093/jscr/rjad730

**Published:** 2024-02-06

**Authors:** Iason Psilopatis, Matthias W Beckmann, Julius Emons, Carla E Schulmeyer

**Affiliations:** Department of Gynecology, University Hospital Erlangen, Universitätsstraße 21/23, 91054 Erlangen, Germany; Department of Gynecology, University Hospital Erlangen, Universitätsstraße 21/23, 91054 Erlangen, Germany; Department of Gynecology, University Hospital Erlangen, Universitätsstraße 21/23, 91054 Erlangen, Germany; Department of Gynecology, University Hospital Erlangen, Universitätsstraße 21/23, 91054 Erlangen, Germany

**Keywords:** hydrosalpinx, ureterocele, infertility

## Abstract

Hydrosalpinx is a common condition in women of reproductive age that correlates with infertility. A ureterocele is a dilatation of the terminal ureter within the bladder and/or urethra that occurs seldomly in adults, but can sonographically be mistaken for a hydrosalpinx. We report of a 29-year-old patient (G2/P1) who was referred in our department with secondary infertility and suspicion of hydrosalpinx. Intraoperatively, no hydrosalpinx could be visualized. Postoperatively, an intravesical cystic mass was detected, alongside a second-degree urinary retention. Sonographically, a double kidney on the left side and an accentuated calyx system could be diagnosed. Ureteroceles seem to represent a rare but still possible differential diagnosis in suspected hydrosalpinx, given the similar sonographic presentation of both conditions.

## Introduction

A ureterocele Is a dilatation of the terminal ureter within the bladder and/or urethra. It is an ectopic form of the distal portion of the ureter that extends to the bladder, bladder neck, or urethra [1]. Ureteroceles may be associated with single or double renal calices. Approximately 80% of ureteroceles are associated with a double collecting system and originate in the upper pole of the ureter. These are ectopias in 60% of cases [2]. Ureteroceles are divided into two classes: intravesical ureteroceles, which are located entirely within the bladder, and ectopic ureteroceles, some of which extend beyond the bladder neck into the urethra [3]. Duplication of the ureters is the most common malformation of the urinary tract at a young age. Females are more commonly affected than males [4, 5]. In women, complete duplex systems have a higher prevalence of vesicoureteral reflux and associated abnormalities [4], such as an obstructed ectopic ureter, pyelonephritis or a ureterocele [6]. Ureteroceles are not all that rare, with an incidence of approximately 1:500 to 1:12000 and are usually asymptomatic [7]. Symptomatic ureteroceles that occur in adulthood are usually difficult to detect and may be associated with secondary complications such as recurrent urinary tract infections.

## Case report

**Figure 1:**
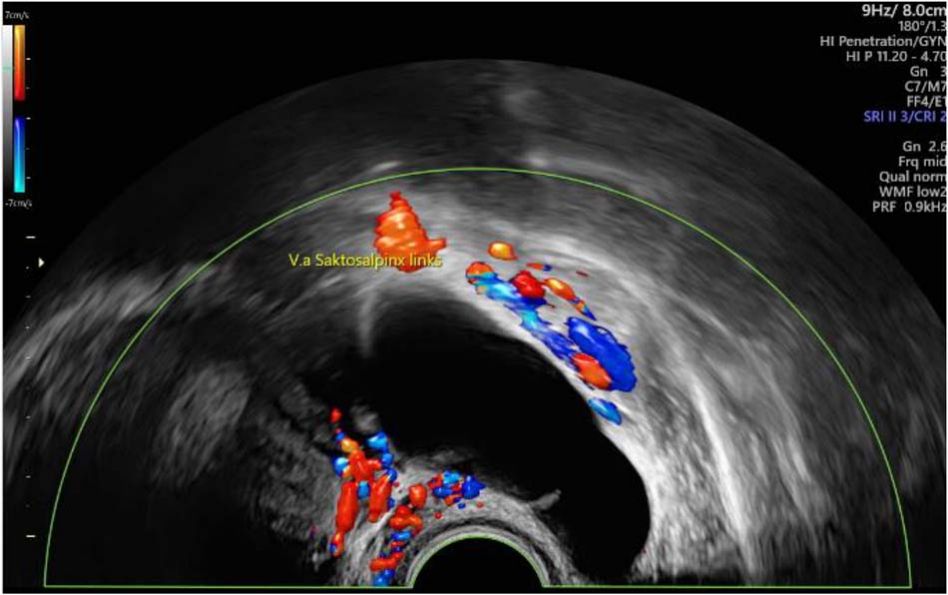
Preoperative sonographic suspicion of hydrosalpinx on the left side (64 × 26 mm^2^, echo-poor with dorsal sound enhancement).

We report of a 29-year-old patient (G2/P1) who was referred in our department with secondary infertility and suspicion of hydrosalpinx for further diagnosis. The patient had no previous diseases, did not take medication regularly and had a regular menstrual cycle. The patient had undergone surgical laparoscopy with salpingotomy on the right side for extrauterine pregnancy. Preoperatively, there was the sonographic suspicion of hydrosalpinx on the left side (64 × 26 mm^2^, echo-poor with dorsal sound enhancement) and of a simple ovarian cyst of the left ovary (Fig. 1). Otherwise, sonography revealed unremarkable internal genitalia, no free fluid in the Douglas space. Hysteroscopy, chromopertubation, laparoscopy and cyst extirpation on the left and, if necessary, salpingectomy on the left were discussed with the patient.

On intraoperative vaginal examination, the vulva and vagina were unremarkable, while the cervix was vulnerable with ectopy. The uterus was 9 cm, mobile, anteverted, anteflexed. Adnexa and parametria bilaterally were unremarkable palpatorily. Hysteroscopically, the cervical canal and cavum were unremarkable. Both openings of the fallopian tubes were macroscopically inconspicuous.

**Figure 2:**
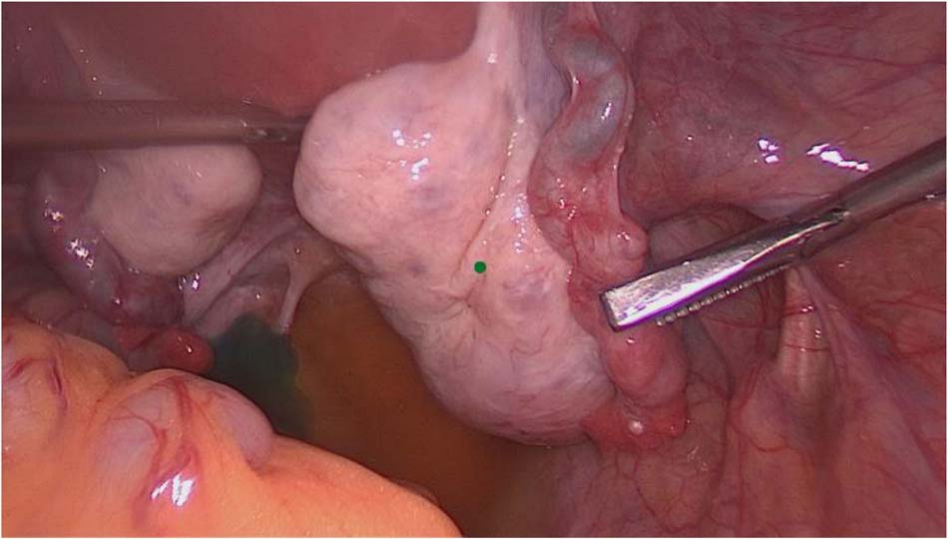
Intraoperative view of the right adnexa (condition after salpingotomy for extrauterine pregnancy).

**Figure 3:**
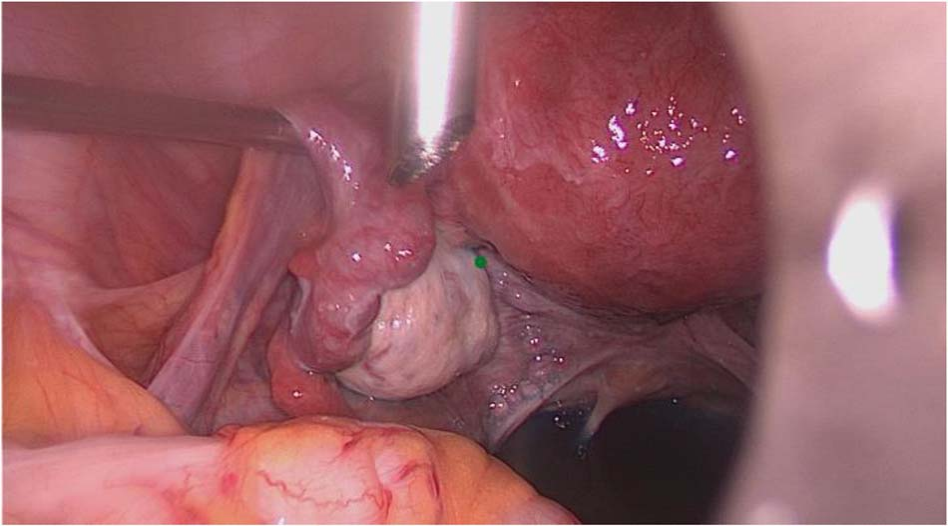
Intraoperative view of the left adnexa.

On laparoscopy, the upper abdominal organs were unremarkable. The uterus was anteverted, anteflexed, with an uneven hyperemic surface, consistent with adenomyosis uteri. The right ovary presented with small cysts, otherwise unremarkable. The right tube appeared bipartite in condition after salpingotomy for extrauterine pregnancy (Fig. 2). The left adnexa was unremarkable without hydroalpinx or ovarian cyst (Fig. 3). The Douglas space was filled with little free fluid. There were no foci of endometriosis in the Douglas space, the ovarian fossae bilaterally, at the bladder envelope fold, or at the bowel. There were no adhesions.

Incision of the ovarian cyst on the right side with the monopolar needle and sampling of the overlay in the fundus area on the left side of the uterus was performed.

Chromopertubation showed both tubes to be promptly open.

**Figure 4:**
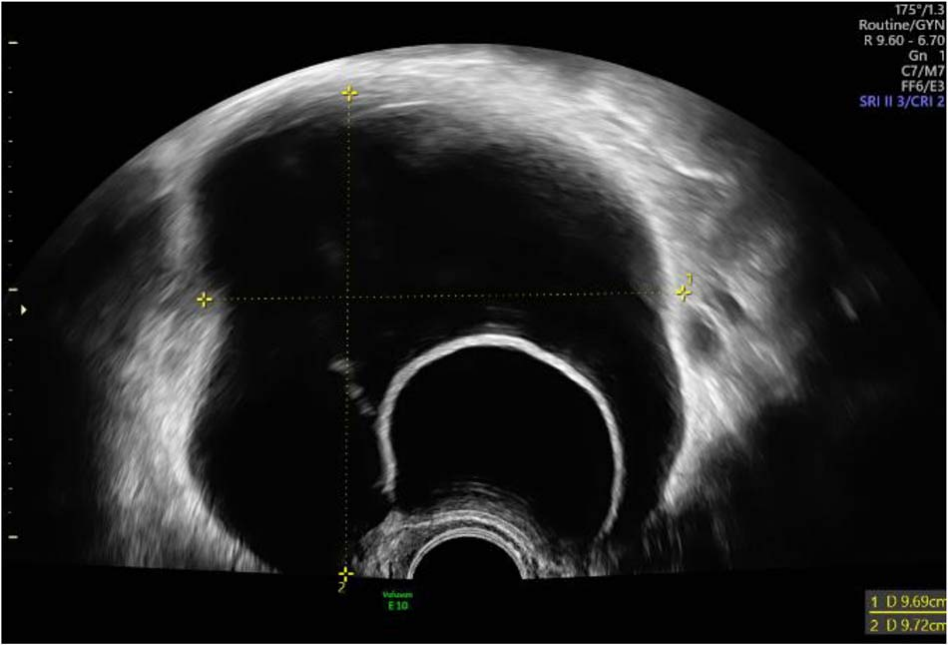
Intravesical cystic mass in the area of the urinary bladder on the left.

The postoperative course was unremarkable. The postoperative laboratory findings were within the normal range. Before discharge, the kidneys and the urinary bladder were sonographically checked as standard procedure for exclusion of intraoperative ureter injury. In the area of the urinary bladder on the left, an intravesical 51 × 43 mm^2^ cystic, smooth bounded mass without suspicious perfusion was detected (Fig. 4). This probably corresponded to the finding described preoperatively as an ovarian cyst. The right kidney showed no evidence of congestion, while on the left side a second-degree urinary retention was diagnosed. Therefore, the patient was consulted by a urologist. Sonographically, a double kidney on the left side with a cyst at the upper pole of the upper part and an accentuated calyx system could be diagnosed.

Pathologic review of the specimen collection of the overlay in the fundal area to the left of the uterus described vascular connective tissue and nodular smooth muscle tissue without significant cytologic atypia or appreciable mitotic activity, corresponding to a small subserosal leiomyoma.

## Discussion

Hydrosalpinx is an accumulation of fluid in the ampullary lumen as a result of occlusion of the infundibulum. It is a common condition in women of reproductive age and is associated with a decreased pregnancy rate [8]. The gold standard for hydrosalpinx diagnosis is histological confirmation, although laparoscopic direct observation is also an accepted method [9]. Non-invasive techniques such as ultrasound have been proposed for diagnosis to achieve adequate assessment of reproductive capacity while avoiding unnecessary laparoscopies. Since a fluid-filled tube is theoretically visible as a cystic mass on transvaginal ultrasound, a hydrosalpinx can be classically identified sonographically [10]. As for the most important sonographic features for an accurate diagnosis of hydrosalpinx, this lesion is described as a typically elongated cystic mass with echo free or hypoechogenic contents, convoluted and with incomplete septa due to ballooning and duplication of the tube. These incomplete septa arise as triangular, hyperechogenic wall protrusions into the lumen that do not reach the opposite wall [11].

Most ureteroceles are diagnosed in utero or immediately after birth during the second or third trimester sonographic screening for renal malformations. Sonographically, a clearly demarcated cystic intravesical mass arising from the posterior bladder wall that can be traced into a dilated distal ureter is characteristic of a ureterocele [2]. However, if the bladder is overdilated, the ureterocele may collapse and not be sonographically visualizable. Only a dilated distal ureter may be seen entering the bladder. If the bladder is inadequately filled, a large ureterocele may occupy the bladder cavity and be mistaken for a partially filled bladder without a ureterocele [12].

In summary, the presented case represents an interesting case of a ureterocele first diagnosed in adulthood with a double kidney, which was misinterpreted as a hydrosalpinx due to the image-morphological correlation in the preoperative ultrasound. Thus, a ureterocele seems to be a rare but nevertheless possible differential diagnosis in suspected hydrosalpinx.
